# Efficiency of Classical and Quantum Games Equilibria

**DOI:** 10.3390/e23050506

**Published:** 2021-04-22

**Authors:** Marek Szopa

**Affiliations:** Department of Operations Research, University of Economics in Katowice, Bogucicka 3, 40-287 Katowice, Poland; marek.szopa@uekat.pl

**Keywords:** game theory, quantum games, Nash equilibrium, Pareto-efficiency, correlated equilibria

## Abstract

Nash equilibria and correlated equilibria of classical and quantum games are investigated in the context of their Pareto efficiency. The examples of the prisoner’s dilemma, battle of the sexes and the game of chicken are studied. Correlated equilibria usually improve Nash equilibria of games but require a trusted correlation device susceptible to manipulation. The quantum extension of these games in the Eisert–Wilkens–Lewenstein formalism and the Frąckiewicz–Pykacz parameterization is analyzed. It is shown that the Nash equilibria of these games in quantum mixed Pauli strategies are closer to Pareto optimal results than their classical counter-parts. The relationship of mixed Pauli strategies equilibria and correlated equilibria is also studied.

## 1. Introduction

Game theory analyzes and models the behavior of agents in the context of strategic thinking and interactive decision making. It is essential in making choices and considering opportunities in business and in everyday life. Examples of situations requiring strategic thinking can be found in economics [1], political science [2], biology [3,4] or military applications [5]. The participating sites have their own sets of possible actions, called strategies, and have preferences over these actions defined by the payoff matrix. Game theory deals with modeling these activities and searching for optimal strategies. Among all notions of game theory, the concept of Nash equilibrium plays a major role. It describes the optimal decisions with regard to the moves of other players. In a Nash equilibrium no player has anything to gain by changing only his own strategy [6].

Game theory results favorable to the whole group of players are called Pareto-efficient. From an economic point of view, they are the most desirable results. However, in many cases, what is beneficial individually is not always also Pareto-efficient. It is often the opposite—striving to meet one’s own interests does not lead to the best solution for all players. This type of dilemma occurs in many real situations regarding e.g., traffic organization [7], excessive exploitation of natural resources [8] or public procurement regulation [9].

Quantum mechanics is one of the most prolific theories of all time. Despite the many controversies it has aroused since the dawn of its history, its predictions have been confirmed experimentally with incredible accuracy. One of the fields that uses the quantum mechanics formalism is quantum economics—a very promising novel field of its application [10,11]. The impetus for the development of this field was the emergence of programmable quantum computers [12]. The various areas of quantum economics research include: market games [13], duopoly problems [14,15], auctions and competitions [16], gambling [17], quantum money [18], quantum annealing [19], quantum cryptography and security issues [20,21] quantum optimal transport [22] or even high-frequency trading [23]. An important role in economic applications is also played by the concept of the probability amplitude utilized by quantum statistics [24].

The purpose of this work is to analyze game mechanisms, that allow players to regulate their choices in such a way that, attempting to optimize their individual interests, they do not create a disadvantage for the group. In the language of game theory, we will strive to reformulate games in such a way that the participants act individually in a favorable manner, i.e., achieve the Nash equilibrium state, and at the same time obtain results as close as possible to the Pareto-efficient results for the group.

Quantum game theory allows to study interactive decision making by players with access to quantum technology. This technology can be used in both of two ways: as a quantum communication protocol and as a way to randomize players’ strategies more efficiently than in classical games [25]. Better randomization of game results by quantum strategies is the key to achieving Pareto-efficient solutions. In this paper we use the Eisert–Wilkens–Lewenstein (EWL) quantization protocol [26], which is the most studied protocol in games of quantum communication. In the EWL approach with the SU(2) strategy set, obtaining Pareto-efficient solutions is feasible but the problem is that this 3-parameter strategy space yield only trivial Nash equilibria. On the other hand many authors tried to investigate EWL scheme with a 2-parameter strategy space in which non-trivial equilibria can be obtained. This, however, leads to an undesirable dependence of the equilibria on the selected parameterization [27]. To resolve this dilemma, we propose using the criterion of quantum game invariance under isomorphic transformations of the input classic game introduced by Frąckiewicz [28]. This criterion allow the full SU(2) strategy parameter space but also selected 2-parameter strategy spaces. On this basis, we build mixed quantum strategies, which yield non-trivial NE and, at the same time, are not arbitrarily chosen parameter subspaces.

We study four games in which the problem of suboptimal Nash’s equilibrium arises: the prisoner’s dilemma, battle of the sexes and two versions of the game of chicken. Thanks to the use of mixed quantum strategies, we obtain both: non-trivial Nash equilibria and that they are closer to Pareto-efficient solutions than classical equilibria. The ultimate goal is to design a quantum device, the input of which is operated by players, parties to the conflict, economic institutions, and the output, through the collapse of the wave function, determines the result of the game, the solution of the dispute or conflict between the parties. The speed with which quantum technologies are currently developing allows us to assume that the efficient quantum strategies may soon be applicable to real practical problems [29].

In the second section, the basic concepts of games and their payouts in pure, mixed strategies and general probability distributions are defined. We also define the concepts of the Nash equilibrium, Pareto-efficiency and correlated equilibrium. The third section, presents four classical games, discuss their Nash equilibria and analyzes their Pareto-optimality. We also discuss their correlated equilibria, which thanks to the use of additional mechanisms of correlation of players’ behavior, allow for better Pareto optimization of the results of these games. The fourth section is devoted to defining the concept of quantum game in the EWL scheme with the full SU(2) parameter space and in the Frąckiewicz Pykacz parameterization. Part five of the paper presents our proposals for new Nash equilibria in quantum mixed strategies and their comparison with correlated equilibria. In the last part we discuss the applicability of both correlation mechanisms and the perspective of physical implementation of quantum games.

## 2. Game Theory Preliminaries

Let us consider a two player, two strategy game $G=\left(N,\;{\left\{S_{X}\right\}}_{X\epsilon N},{\left\{P_{X}\right\}}_{X\epsilon N}\right)$, where N={A,B} is the set of players (Alice and Bob), $S_{A}=\left\{A_{0},A_{1}\right\}$, $S_{B}=\left\{B_{0},B_{1}\right\}$ are sets of their possible *pure strategies* (or actions) and $P_{X}:S_{A}\times S_{B}\to \{v_{ij}^{X}\;\epsilon \;\mathbb{R}\;|\;i,j=0,1\},$ are respective payoff functions for Player X, X=A,B, usually represented by a game bimatrix $\left(\begin{array}{cc} \left(v_{00}^{A},v_{00}^{B}\right) & \left(v_{01}^{A},v_{01}^{B}\right)\\ \left(v_{10}^{A},v_{10}^{B}\right) & \left(v_{11}^{A},v_{11}^{B}\right) \end{array}\right)$. Let us denote by

(1) $$\Delta \left(S_{A}\times S_{B}\right)=\left\{\sum_{i,j=0,1}\sigma_{ij}A_{i}B_{j}|\;\sigma_{ij}\geq 0,\sum_{i,j=0,1}\sigma_{ij}=1\right\}$$

the set of all probability distributions over $S_{A}\times S_{B}$. The payoff of a Player X, corresponding to a given distribution $\sigma ={\left\{\sigma_{ij}\right\}}_{i,j=0,1}$ is

(2) $$\Delta P_{X}\left(\sigma \right)=\underset{i,j=0,1}{\sum }\sigma_{ij}v_{ij}^{X}$$

Let us now restrict the set of all probability distributions to distributions, that can be factorized, i.e., presented in a form

(3) $$\left(\begin{array}{cc} \sigma_{00} & \sigma_{01}\\ \sigma_{10} & \sigma_{11} \end{array}\right)=\left(\begin{array}{cc} \sigma_{A}\sigma_{B} & \sigma_{A}\left(1-\sigma_{B}\right)\\ \left(1-\sigma_{A}\right)\sigma_{B} & \left(1-\sigma_{A}\right)\left(1-\sigma_{B}\right) \end{array}\right)$$

They define mixed strategy spaces

$$\Delta S_{X}\equiv \Delta \left(S_{X}\right)=\{\sigma_{X}X_{0}+\left(1-\sigma_{X}\right)X_{1}\;|\;0\leq \sigma_{X}\leq 1\}\equiv \left[0,1\right],\;\;X=A,B$$

which are defined by a single number $\sigma_{X}\;\epsilon \;\left[0,1\right]$. Note that the product of mixed strategy spaces is a subset of the set of all probability distributions ${\Delta S}_{A}\times {\Delta S}_{B}\subset \Delta \left(S_{A}\times S_{B}\right)$.

Given a profile $\sigma =\left(\sigma_{A},\sigma_{B}\right)\;\epsilon \;{\Delta S}_{A}\times {\Delta S}_{B}$ of mixed strategies of both players, Player X obtains an expected payoff which is an element of Δ(ImPX)—the set of probability distributions over the outcomes of G. It leads to the notion of the *mixed classical game* $G^{mix}=\left(N,\;{\Delta S}_{A},{\Delta S}_{B},{\Delta P}_{A},{\Delta P}_{B}\right)$, where payoffs ΔPX:[0,1]×[0,1]→Δ(ImPX) are defined by (2) and (3).

Let us define a vector valued payoff function $\Delta P:\Delta \left(S_{A}\times S_{B}\right)\to {\mathbb{R}}^{2}$ by $\Delta P\left(\sigma \right)=\left({\Delta P}_{A}\left(\sigma \right),{\Delta P}_{B}\left(\sigma \right)\right)$. The range of the payoff function of the mixed game is $R_{G^{mix}}=\Delta P\left(\Delta_{A}\times \Delta_{B}\right)$. The range of all probability distributions (1) over $S_{A}\times S_{B}$ is $R_{PD}=\Delta P\left(\Delta \left(S_{A}\times S_{B}\right)\right)$. Note that $R_{G^{mix}}$ is usually a proper subset of the range of all probability distributions $R_{G^{mix}}\subset R_{PD}$.

The pair of strategies $\left(\sigma_{A}^{*},\sigma_{B}^{*}\right)\epsilon {\Delta S}_{A}\times {\Delta S}_{B}$ is a Nash equilibrium (NE), if for each strategy $\sigma_{X}\epsilon {\Delta S}_{X}$, X=A,B, ${\Delta P}_{A}\left(\sigma_{A}^{*},\sigma_{B}^{*}\right)\geq {\Delta P}_{A}\left(\sigma_{A},\sigma_{B}^{*}\right)$ and ${\Delta P}_{B}\left(\sigma_{A}^{*},\sigma_{B}^{*}\right)\geq {\Delta P}_{B}\left(\sigma_{A}^{*},\;\sigma_{B}\right)$, i.e., no player has a profitable unilateral deviation from his strategy, while the other stays with his [30]. Thus, NE is such a pair of players’ strategies for which they all achieve their optimal (for a given strategy of other player) individual efficiency. In the same way one can define, that a pair $\left(A^{*},B^{*}\right)\;\epsilon {\;S}_{A}\times S_{B}$ is a Nash equilibrium of the (pure) game G, if for each strategy $X_{i}\;\epsilon {\;S}_{X}$, X=A,B, $P_{A}\left(A^{*},B^{*}\right)\geq P_{A}\left(A_{i},B^{*}\right)$ and $P_{B}\left(A^{*},B^{*}\right)\geq P_{B}\left(A^{*},\;B_{i}\right)$. Whereas the celebrated Nash’s theorem says that every mixed classical game has a Nash equilibrium (in mixed strategies), it does not have to be true for every (pure) game G [31].

From the viewpoint of mutual efficiency, the concept of Pareto optimality plays an important role. Let S be an arbitrary set of strategies. A pair of strategies $\left(\sigma_{A},\sigma_{B}\right)\;\epsilon \;S$ is *not Pareto optimal* in S if there exists another pair, $\left({\sigma_{A}}^{\prime },{\sigma_{B}}^{\prime }\right)\;\epsilon \;S$ that is better for one of the players ${\Delta P}_{X}\left(\sigma_{A},\sigma_{B}\right)<{\Delta P}_{X}\left({\sigma_{A}}^{\prime },{\sigma_{B}}^{\prime }\right)$, and not worse for the other Player ${\Delta P}_{-X}\left(\sigma_{A},\sigma_{B}\right)\leq {\Delta P}_{-X}\left({\sigma_{A}}^{\prime },{\sigma_{A}}^{\prime }\right)$, where −X is the remaining player for player X=A,B, otherwise the pair $\left(\sigma_{A},\sigma_{B}\right)$ is called *Pareto optimal* (or *Pareto-efficient*) in S. A set of all Pareto optimal strategies for a given set of strategies S is called the *Pareto frontier of* S and denoted 𝒫O(S). For instance a pair of strategies $\left(\sigma_{A},\sigma_{B}\right)\;\epsilon \;{\Delta S}_{A}\times {\Delta S}_{B}$ is Pareto optimal in ${\Delta S}_{A}\times {\Delta S}_{B}$ if there exist no other set of mixed strategies, that would be better for at least one of players and not worse for the other. Note that the Pareto optimal strategy in a set S is not necessarily optimal in a larger set $S^{\prime }⊃S$.

An interesting concept of optimizing equilibria beyond the classical game theory was put forward by R. Aumann. By correlated equilibrium, we understand a situation in which players make their optimal decisions, guided by an external signal, transmitted to them by a trusted correlating device according to a given probability distribution. Each player maximizes his expected payoff by following this recommendation. Formally, probability distribution ${\left\{\sigma_{ij}\right\}}_{i,j=0,1}$ over the set of action vectors ${\left(A_{i},B_{j}\right)}_{i,j=0,1}$ of the game G is called a *correlated equilibrium* [31], if for every strategy $A_{i}\epsilon S_{A}$ and $B_{i}\epsilon S_{B}$

(4) $$\underset{j=0,1}{\sum }\sigma_{ij}v_{ij}^{A}\geq \underset{j=0,1}{\sum }\sigma_{ij}v_{-ij}^{A}\;\mathrm{and}\;\underset{j=0,1}{\sum }\sigma_{ji}v_{ji}^{B}\geq \underset{j=0,1}{\sum }\sigma_{ji}v_{j\left(-i\right)}^{B}$$

respectively, where $X_{-i}\epsilon S_{X}$ is the remaining strategy −i≠i.

One of the advantages of correlated equilibria is that they are computationally easier than Nash equilibria. Computing the correlated equilibrium requires only solving the linear problem, while solving the Nash equilibrium requires solving the equations that make each player’s payoffs independent of the others.

## 3. The Efficiency of Selected Classical Games

The most contrasting example of the lack of Pareto optimality for Nash equilibria is the prisoner’s dilemma (PD) game [32]. The game is universal in nature and describes many decision-making dilemma commonly found in different situations of social life. It is defined by $\mathrm{PD}=\left(N,\;{\left\{S_{X}\right\}}_{X\epsilon N},{\left\{P_{X}\right\}}_{X\epsilon N}\right)$ and the payoffs are defined by the bimatrix in Table 1, where t>r>p>s and $r>\frac{s+t}{2}$ [33]. A typical scenario assumes that two players, Alice and Bob, independently of each other, choose one of two strategies—“cooperation” $A_{0}$ and $B_{0}$ or “defection” $A_{1}$ and $B_{1}$.

It is easy to see that regardless of the opponent’s choice, the dominant strategy of each player is to “defect” and the pair of mutual defection strategies ($A_{1}$,$B_{1}$) is the Nash equilibrium of the game. On the other hand the Pareto-efficient solutions are all the remaining pairs of pure strategies. Moreover, when allowing the players to randomize their strategies, the Nash equilibrium remains the same and the Pareto frontier of ${\Delta S}_{A}\times {\Delta S}_{B}$ is $A_{0}\times {\Delta S}_{B}\cup {\Delta S}_{A}\times B_{0}$. In case of typical game payoffs: t=5, r=3,p=1, s=0, the Nash equilibrium ($A_{1}$,$B_{1}$) with a payoff of (1,1) is far from the Pareto optimal ($A_{0},B_{0}$) with a payoff of (3,3). One can show that the only correlated equilibrium (4) of PD is of the form $\sigma_{\mathrm{PD}}=\left(\begin{array}{cc} 0 & 0\\ 0 & 1 \end{array}\right)$, i.e., coincides with its NE and does not improve Pareto efficiency. It is because both cooperation strategies $A_{0}$ and $B_{0}$ are strictly dominated and therefore can never be played in a correlated equilibrium.

The second game under consideration is battle of the sexes (BoS), defined by the payoff bimatrix in Table 2. Alice and Bob plan to be together, for which they can get paid 2. However, Alice would prefer to go to the theater $X_{0}$, whereas Bob would prefer the football game $X_{1}$, X=A,B. Going to a preferred place gives players an additional bonus of + 1.

This game has two Nash equilibria $\left(A_{0},B_{0}\right)$ and $\left(A_{1},B_{1}\right)$ in pure strategies. Both of them form a set of Pareto optimal solutions $𝒫O\left({\Delta S}_{A}\times {\Delta S}_{B}\right)=\left(A_{0},B_{0}\right)\cup \left(A_{1},B_{1}\right)$ but the problem, which gives the name to the game, is that they can not be both satisfied with a just solution. One player consistently does better than the other. BoS has also one NE in mixed strategies, in which players go to their preferred event more often than the other. It is given by a pair of strategies $\sigma_{A}=\frac{3}{4}A_{0}+\frac{1}{4}A_{1}$, $\sigma_{B}=\frac{1}{4}B_{0}+\frac{3}{4}B_{1}$, for Alice and Bob, respectively. The mixed strategy NE, where they both get the same payoff $\left(\frac{3}{2},\frac{3}{2}\right)$ is however not Pareto-efficient even in ${\Delta S}_{A}\times {\Delta S}_{B}$ because e.g., each of the pure strategy NE is better for both players. One can also find an correlated equilibrium for this game, that according to (4) are defined by inequalities: $3\sigma_{00}\geq \sigma_{01}$, $\sigma_{00}\geq 3\sigma_{10}$, $3\sigma_{11}\geq \sigma_{01}$ and $\sigma_{11}\geq 3\sigma_{10}$. The Pareto frontier of correlated equilibria is the set $\left\{\left(\begin{array}{cc} \sigma & 0\\ 0 & 1-\sigma \end{array}\right)|0\leq \sigma \leq 1\right\}$ and equal payoff optimal solution is then $\left(2\frac{1}{2},2\frac{1}{2}\right)$, achievable for the distribution $\sigma_{\mathrm{BoS}}=\left(\begin{array}{cc} 1/2 & 0\\ 0 & 1/2 \end{array}\right)$. It means that the players go together to the theater or the game depending on the coin toss. This payoff is higher than the Nash equilibrium in mixed strategies and is Pareto-optimal $\sigma_{\mathrm{BoS}}\epsilon 𝒫O(\Delta (S_{A}\times S_{B}))$ in the set of all probability distributions, moreover it is not accessible by any mixed strategy $\sigma_{\mathrm{BoS}}\notin \;{\Delta S}_{A}\times {\Delta S}_{B}$.

The last of the classical games we consider is the game of chicken CG (chicken game), with the payoff bimatrix defined by Table 3. This game describes, e.g., the behavior of two drivers approaching, one from the south and the other from the west, at the same time to the intersection. They both have two options: to cross the intersection $X_{1}$ or to stop $X_{0}$ before it, X=A,B. If both of them choose the option to drive, they will collide and both lose 10. If only one of them passes and the other stops, the passing one wins (1,0). If both of them stop, the result is neutral (0,0).

CG has two Nash equilibria in pure strategies $\left(A_{0},B_{1}\right)$ and $\left(A_{1},B_{0}\right)$, which are Pareto-efficient. However, none of these equilibria, just like in BoS, satisfy both players. The game also has the third equilibrium in mixed strategies: each car passes a crossroads with a probability of 1/11. This equilibrium is fair—both players receive equal payouts, but the trouble is that both payouts are equal to 0, and therefore not optimal in ${\Delta S}_{A}\times {\Delta S}_{B}$—each player can increase his payout by increasing the frequency of crossing, while the other stops at the junction. The Pareto frontier of correlated equilibria (4) is the set $\left\{\left(\begin{array}{cc} 0 & \sigma \\ 1-\sigma & 0 \end{array}\right)|0\leq \sigma \leq 1\right\}$ and the equal payoff correlated equilibrium is $\sigma_{\mathrm{CG}}=\left(\begin{array}{cc} 0 & 1/2\\ 1/2 & 0 \end{array}\right)$, i.e., each of the drivers passes the intersection with a probability of ½ while the other one stops. Such a solution is realized by traffic lights. It is a correlated equilibrium because none of the drivers is interested in running a red light, knowing that the other one is green at that time. If they both comply with the traffic rules, they will receive a payment of ½, i.e., higher than the mixed strategy Nash equilibrium. It has the highest, equal for both players payoff because it is Pareto-efficient in the set of all probability distributions $\sigma_{\mathrm{CG}}\epsilon 𝒫O(\Delta (S_{A}\times S_{B}))$ but not accessible by any mixed strategy as $\sigma_{\mathrm{CG}}\notin \;{\Delta S}_{A}\times {\Delta S}_{B}$.

The last game we will consider is another version of the chicken game (Table 4):

As in the previous game, the winner is the player who chooses the $X_{1}$ option while the other one plays $X_{0}$, X=A,B. The best fair solution is for both players to choose $\left(A_{0},\;B_{0}\right)$ but it is not an equilibrium. As before, this game has three Nash equilibria: two in pure strategies $\left(A_{1},B_{0}\right)$ and $\left(A_{0},B_{1}\right)$ and one in a mixed strategy, in which both players choose $X_{0}$ and $X_{1}$ with equal probabilities $\sigma_{X}=\frac{1}{2}X_{0}+\frac{1}{2}X_{1}$, X=A,B. The payoffs for these Nash equilibria are: (5,1), (1,5) and (2$\frac{1}{2},\;2\frac{1}{2}$) respectively. As before, Pareto-efficient equilibria are not fair (in the sense that one player wins and the other loses), and the fair equilibrium is not Pareto-efficient (because both players can score better in ${\Delta S}_{A}\times {\Delta S}_{B}$ by choosing $\left(A_{0},B_{0}\right)$. It follows from (4) that the correlated equilibrium for this game should obey four inequalities: $\sigma_{00}\leq \sigma_{01}$, $\sigma_{00}\leq \sigma_{10}$, $\sigma_{11}\leq \sigma_{01}$ and $\sigma_{11}\leq \sigma_{10}$. Therefore the Pareto frontier of the set of correlated equilibria is

$$\left\{\left(\begin{array}{cc} \sigma^{\prime } & \sigma \\ 1-\sigma -\sigma^{\prime } & 0 \end{array}\right)|\;0\leq \sigma \leq 1,\;\sigma^{\prime }=\max\left(\sigma ,\;\frac{1-\sigma }{2}\right)\;\right\}$$

and the maximal symmetric payoff is ($3\frac{1}{3},\;3\frac{1}{3}$), corresponding to $\sigma_{\mathrm{CG}2}=\left(\begin{array}{cc} 1/3 & 1/3\\ 1/3 & 0 \end{array}\right)$. It is better than the symmetric Nash equilibrium.

Aumann [34] proposed the following mechanism of correlated equilibrium realization. Let’s consider the third side (or some natural event), which with a probability of 1/3 draws one of three cards marked: (0,0), (0,1) and (1,0). After the card is drawn, the third party informs the players about the strategy assigned to them on the card (but not about the strategy assigned to the opponent). Suppose one player is assigned “1”, knowing that the other player saw “0” (because there is only one card that assigns him “0”), he should play “1” because he will receive the highest possible payout 5. Let’s assume that the player was assigned “0”. Then he knows, that the other player has received “0” or “1” commands, with probabilities 1/2. The expected payoff for playing “1” (contrary to the recommendation) is therefore $5\times \frac{1}{2}+0\times \frac{1}{2}=\frac{5}{2}$, and the expected payout for playing as recommended “0” is the same $4\times \frac{1}{2}+1\times \frac{1}{2}=\frac{5}{2}$. Because none of the players has motivation to play differently than was recommended by the third party, the result of the draw is the correlated equilibrium. The probability distribution $\sigma_{\mathrm{CG}2}\;\epsilon \;\Delta \left(S_{A}\times S_{B}\right)$ can not be factorized as in Equation (3) and therefore is not a mixed game strategy $\sigma_{\mathrm{CG}2}\notin \;{\Delta S}_{A}\times {\Delta S}_{B}$. It is also not Pareto-efficient $\sigma_{\mathrm{CG}2}\notin 𝒫O(\Delta (S_{A}\times S_{B}))$ in the set of all probability distributions.

The disadvantage of correlated equilibria is the need to use an external signal that must be generated by an independent device that can be manipulated. Therefore, it is worth looking for correlation mechanisms that would be safe and not susceptible to manipulation. As in the field of cryptography [35], such a solution may be transferring games to the quantum domain.

## 4. EWL Quantization Protocol in Frąckiewicz–Pykacz Parameterization

In recent years, we have witnessed the rapid development of research on quantum information processing [36,37] and successful experiments related to the engineering of entangled qubits [38,39]. In the laboratories of Google Quantum AI [12], IBM [40], D-wave and several other companies [41], there is a race to achieve the so-called quantum supremacy. Google AI Quantum managed to construct a quantum processor based on 53 qubits, which in 200 s solved a problem that a classical computer would solve in 10 thousand years [12]. In the field of possible applications of quantum engineering, quantum games are also attracting much attention [42,43]. Apart from their own intrinsic interest, quantum games explore the fascinating world of quantum information [44,45,46].

The idea of using quantum computers to extend classical games to the quantum domain was put forward at the end of the 20th century. In his groundbreaking work on the theory of quantum games [47], Meyer proposed a simple coin toss game and showed that a player using quantum superposition will always win against a classical player. A general protocol for quantum games was proposed by Eisert, Wilkens and Lewenstein (EWL) [26]. This model has been widely discussed [48] and, e.g., extended to multiplayer games [49].

In this approach, players’ strategies are operators in a certain vector space known as a *Bloch sphere* [50]. This space is a set of *qubits*—normalized vectors with complex coefficients spanned on a two-element basis {|0⟩, |1⟩} which, up to the phase, can be represented in the form

(5) $$\left|\psi \right\rangle =\cos\frac{\theta }{2}|0\rangle +e^{i\varphi }\sin\frac{\theta }{2}\left|1\right\rangle$$

where θ∈[0,π] and φ∈[−π,π]. An example of the qubit can be any quantum mechanical two-state system such as an electron with spin up or down, or a photon in two different polarizations.

Qubits |ψ⟩ representing a superposition of the basis states |0⟩ and |1⟩ are pure quantum states. A qubit in a state (5) does not have any value “between” |0⟩ and |1⟩. It means that before the measurement is carried out, it is not defined and only the measurement yields a value of |0⟩ or |1⟩ with probabilities $\cos^{2}\frac{\theta }{2}$ and $\sin^{2}\frac{\theta }{2}$ respectively. This process is called the collapse of the wave function. For example, all qubits representing states with θ=π/2, i.e., at the equator of the Bloch sphere represent a quantum state which, after measurement, collapses to the state |0⟩ or |1⟩ with probabilities equal to $\frac{1}{2}$.

Now let us consider a space of qubit pairs, one for each player. In this product space the standard *observational basis* is {|00⟩, |01⟩, |10⟩, |11⟩}, where the first (second) qubit belongs to the first (second) player. Then let’s use the entangling operator $\hat{J}=\cos\left(\frac{\gamma }{2}\right)\hat{I}⊗\hat{I}+i\sin\left(\frac{\gamma }{2}\right)\sigma_{x}⊗\sigma_{x}$, where $\hat{I}$ is the unit operator, $\sigma_{x}=\left(\begin{array}{cc} 0 & 1\\ 1 & 0 \end{array}\right)$ is the Pauli matrix and $\gamma \in \left[0,\frac{\pi }{2}\right]$, represents the entanglement level, to prepare the initial quantum state $\left|\psi_{0}\right\rangle =\hat{J}\left|00\right\rangle$. For γ=0, this state is separable $\left|\psi_{0}\right\rangle =\left|00\right\rangle$, whereas for $\gamma =\frac{\pi }{2}$, the initial state $\left|\psi_{0}\right\rangle =\frac{1}{\sqrt{2}}\left(\left|00\right\rangle +i\left|11\right\rangle \right)$ is the maximally entangled (Bell) state [51]. From now on, we assume that $\gamma =\frac{\pi }{2}$, i.e., the initial state is fully entangled. Quantum entanglement is a nonlocal property that allows a set of qubits to express higher correlation than is possible in classical systems, e.g., if one of the owners of the entangled pair performs a measurement of his part, it immediately determines the result of the measurement of the other party, regardless of how far away they may be. We also assume that the initial entangled state $\left|\psi_{0}\right\rangle$ is known to both players.

From the Schrödinger equation, describing the time evolution of quantum states, it follows that the transformations governing it must be unitary. Therefore, in quantum game theory, players’ strategies are unitary transformations ${\hat{U}}_{A}$ i ${\hat{U}}_{B}$ operating on the initial state $\left|\psi_{0}\right\rangle$. They correspond to the manipulations that are performed by the players, each on its own part of an entangled qubit. Transformations ${\hat{U}}_{X}\in \mathrm{SU}\left(2\right),$ X=A,B are defined by unitary matrices

(6) $${\hat{U}}_{X}\left(\theta_{X},\alpha_{X},\beta_{X}\right)=\left(\begin{array}{cc} e^{i\alpha_{X}}\cos\frac{\theta_{X}}{2} & ie^{i\beta_{X}}\sin\frac{\theta_{X}}{2}\\ ie^{-i\beta_{X}}\sin\frac{\theta_{X}}{2} & e^{-i\alpha_{X}}\cos\frac{\theta_{X}}{2} \end{array}\right)$$

where, $\theta_{X}\in \left[0,\pi \right]$ and $\alpha_{X},\;\beta_{X}\in \left[0,2\pi \right]$, X=A, B. The quantum state obtained in this way is then in the EWL protocol disentangled by the $\hat{J^{†}}$ (Hermitian conjugate of $\hat{J}$) operator. The final state of this operation is

(7) $$\left|\psi_{f}\right\rangle =\hat{J^{†}}\left({\hat{U}}_{A}\;⊗\;{\hat{U}}_{B}\right)\hat{J}\;\left|00\right\rangle$$

and can be expressed in an observational basis by $\left|\psi_{f}\right\rangle =\sum_{i,j=0,1}p_{ij}\;\left|ij\right\rangle$, where ${\left|p_{ij}\right|}^{2}={\left|\langle ij|\psi_{f}\rangle \right|}^{2}$, i,j=0,1 are probabilities that the final state measurement will give one of four vectors in the observational basis.

**Figure 1:**
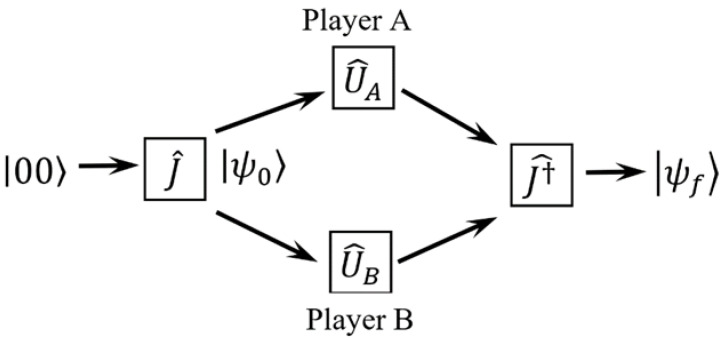
The quantum game in EWL protocol.

The sequence of operations that makes up the quantum game is schematically represented in Figure 1.

The quantum game in the Eisert–Wilkens–Lewenstein protocol is defined as a triple $\Gamma_{EWL}=\left(N,\;{\left\{U_{X}\right\}}_{X\epsilon N},\;{\left\{\Pi_{X}\right\}}_{X\epsilon N}\right)$, where N={A,B} is the set of players, $U_{X}$ are sets of unitary transformations (6) ${\hat{U}}_{X}\in U_{X}$, that are pure strategies of the players and $\Pi_{X}:\mathrm{SU}\left(2\right)\times \mathrm{SU}\left(2\right)\to \mathbb{R}$ is the payoff function defined by

(8) $$\Pi_{X}\left({\hat{U}}_{A},\;{\hat{U}}_{B}\right)=\underset{i,j=0,1}{\sum }{\left|p_{ij}\right|}^{2}v_{ij}^{X},\;\;X=A,B$$

where {$v_{ij}^{X}$} is the payoff bimatrix of the corresponding classical game. In the original formulation of the EWL model, transformations (6) are limited to the two-dimensional parameter space, where $\beta =-\frac{3}{2}\pi$ is constant. However, Benjamin and Hayden [52] observed that the set of 2-parameter quantum strategies is not closed under composition and therefore it seems unlikely, that the restriction can reflect any reasonable physical constraint. A more significant argument has been put forward by Frąckiewicz [28] who showed that this 2-parameter set of strategies may yield different optimal strategy profiles depending on the order of player’s strategies in the classical game. The necessary condition to be satisfied by the parameterization scheme is its invariance under isomorphic transformations of the input game. This condition is met by the full SU(2) strategy parameter space and also by 2-parameter strategy set introduced by Frąckiewicz and Pykacz [53]

(9) $${\hat{V}}_{X}\left(\theta_{X},\phi_{X}\right)=\left(\begin{array}{cc} e^{i\phi_{X}}\cos\frac{\theta_{X}}{2} & ie^{i\phi_{X}}\sin\frac{\theta_{X}}{2}\\ ie^{-i\phi_{X}}\sin\frac{\theta_{X}}{2} & e^{-i\phi_{X}}\cos\frac{\theta_{X}}{2} \end{array}\right),\;\theta_{X}\in \left[0,\pi \right],\;\phi_{X}\in \left[0,2\pi \right]$$

In this parameterization, the observational basis probabilities are:

(10) $$\begin{array}{c} {\left|p_{00}\right|}^{2}={\left(\cos\frac{\theta_{A}}{2}\cos\frac{\theta_{B}}{2}\cos\left(\phi_{A}+\phi_{B}\right)+\sin\frac{\theta_{A}}{2}\sin\frac{\theta_{B}}{2}\sin\left(\phi_{A}+\phi_{B}\right)\right)}^{2}\\ {\left|p_{01}\right|}^{2}={\left(\cos\frac{\theta_{A}}{2}\sin\frac{\theta_{B}}{2}\cos\left(\phi_{A}-\phi_{B}\right)-\sin\frac{\theta_{A}}{2}\cos\frac{\theta_{B}}{2}\sin\left(\phi_{A}-\phi_{B}\right)\right)}^{2}\\ {\left|p_{10}\right|}^{2}={\left(\cos\frac{\theta_{A}}{2}\sin\frac{\theta_{B}}{2}\sin\left(\phi_{A}-\phi_{B}\right)+\sin\frac{\theta_{A}}{2}\cos\frac{\theta_{B}}{2}\cos\left(\phi_{A}-\phi_{B}\right)\right)}^{2}\\ {\left|p_{11}\right|}^{2}={\left(\cos\frac{\theta_{A}}{2}\cos\frac{\theta_{B}}{2}\sin\left(\phi_{A}+\phi_{B}\right)-\sin\frac{\theta_{A}}{2}\sin\frac{\theta_{B}}{2}\cos\left(\phi_{A}+\phi_{B}\right)\right)}^{2}. \end{array}$$

In the special case where the players’ strategies are defined only by the angle θ, with $\phi_{A}=\phi_{B}=0,$ they can be expressed by $\hat{V}\left(\theta ,0\right)=\cos\frac{\theta }{2}\hat{I}+i\sin\frac{\theta }{2}\sigma_{x}$. In this case, $\hat{V}\left(0,0\right)=\hat{I}$ is the unit matrix corresponding to the classical $X_{0}$ strategy and $\hat{V}\left(\pi ,0\right)=\left(\begin{array}{cc} 0 & i\\ i & 0 \end{array}\right)$ is the matrix that is flipping (up to a constant) |0⟩ and |1⟩ qubits and corresponds to the classical $X_{1}$ strategy X=A,B. General 1-parameter strategy $\hat{V}\left(\theta ,0\right)$ is equivalent to the classical mixed strategy for which the probabilities of both pure strategies $X_{0}$ and $X_{1}$ are $\cos^{2}\frac{\theta_{X}}{2}$ and $\sin^{2}\frac{\theta_{X}}{2}$ respectively. In this way the classical game becomes a special case of the quantum game.

Quantum games can be physically implemented by a quantum computer operating according to the above algorithm. Such an algorithm was carried out experimentally [54,55] in EPR-type experiments based on measurements of the Stern Gerlach effect. The players initially share an entangled pure quantum state $\left|\psi_{0}\right\rangle$. Each of them apply his strategy by performing arbitrary local unitary operations on his own qubit, but no direct communication between players is allowed. The result of the game is revealed, by measuring the final state (7) which, as a result of the collapse of the wave function, will give one of the four possible states with the appropriate probability. Due to the fact that players use quantum strategies, entanglement offers opportunities for players to interact with each other, which has no analogue in classical games.

The probability distribution leading to the payoff of the quantum game (8) is, in general, non-factorizable and, therefore, can play a role of the external device correlating player actions proposed by Aumann. There is no need to use cryptographic protocols to replace the trusted mediator [56]. In this case, quantum mechanics offers the possibility of randomizing players’ strategies better than classical methods.

## 5. Efficiency of Quantum Games Equilibria

Let us go back to optimization of game equilibria. For a quantum game so defined, the Nash equilibrium can be defined in exactly the same way as in the classical games. Note however, that the discrete set of pure strategies $S_{X}$ is, in a quantum game replaced by a continuous domain $U_{X}$, which elements depend on 2 or 3 parameters.

For the classical prisoner’s dilemma (Table 1), the only Nash equilibrium is the mutual defection ($A_{1}$, $B_{1}$). In the quantum case and original EWL quantization scheme with 2D parameter space (fixed $\beta =-\frac{3}{2}\pi$), there is a new Nash equilibrium, the “magic” strategy denoted by $\hat{Q}\equiv \hat{U}\left(0,\frac{\pi }{2},-\frac{3}{2}\pi \right)$, corresponding to the Pareto-efficient payoff (3, 3) [26]. However, if we consider the above strategy in the full SU(2) space, then the “Nash equilibrium” obtained in this way ceases to be the equilibrium. Indeed, for any strategy $\hat{U_{A}}\left(\theta ,\alpha ,\beta \right)\in \mathrm{SU}\left(2\right)$, there is a strategy $\hat{U_{B}}=\hat{U}\left(\theta +\pi ,\beta -\pi /2,\alpha \right)$ which “cancels” the action $\hat{U}$ of the Player A and changes the game result to (0, 5) in favor of the Player B. The result is the same if the answer of the Player B is $\hat{{U^{\prime }}_{B}}=\hat{U}\left(\theta +\pi ,\beta +\frac{\pi }{2},\alpha +\pi \right)$. It is then evident, that in the SU(2) case of EWL a Nash equilibrium can exist only in a trivial case, when the original game bimatrix has a result $v_{ij}^{X}$, which is maximal for both players, X=A,B. This conclusion significantly reduces the usefulness of the EWL scheme with a full group of SU(2) strategies for the search for Pareto efficient equilibria. As shown in [53], non-trivial Nash equilibria are also possible in the FP parameterization of an EWL scheme.

In analogy to classical games, Nash equilibria can be defined also for mixed quantum games in mixed quantum strategies [57,58]. Classification of Nash equilibria in mixed strategies for the full SU(2) group of EWL strategies was studied in [59,60]. Here we find mixed strategy equilibria for the FP parameterization of EWL model. Let us consider a set of quantum strategies:

(11) $$\begin{array}{c} \hat{P_{0}}=\hat{V}\left(0,0\right)=\left(\begin{array}{cc} 1 & 0\\ 0 & 1 \end{array}\right),\\ \hat{P_{x}}=\hat{V}\left(\pi ,\pi \right)=\left(\begin{array}{cc} 0 & -i\\ -i & 0 \end{array}\right),\\ \hat{P_{y}}=\hat{V}\left(\pi ,\pi /2\right)=\left(\begin{array}{cc} 0 & -1\\ 1 & 0 \end{array}\right),\\ \hat{P_{z}}=\hat{V}\left(0,\pi /2\right)=\left(\begin{array}{cc} i & 0\\ 0 & -i \end{array}\right). \end{array}$$

The names of these strategies refer to their similarity to the Pauli matrices $\hat{P_{x}}=-i\hat{\sigma_{x}}$, $\hat{P_{y}}=-i\hat{\sigma_{y}}$ and $\hat{P_{z}}=i\hat{\sigma_{z}}$, and therefore can be named Pauli strategies. Although they are generated through a 2-parameter family of operators, they form a basis of infinitesimal generators of the whole SU(2).

Let us consider a quantum game $\Gamma_{EWL}$, where the set of unitary strategies is $U_{X}=\left\{\hat{P_{0}},\hat{P_{x},\;}\hat{P_{y}},\hat{P_{z}}\right\}$. The final state of the game $\left|\psi_{f}\right\rangle =\hat{J^{†}}\left({\hat{P}}_{\alpha }\;⊗\;{\hat{P}}_{\beta }\right)\hat{J}\;\left|00\right\rangle$, where α,β∈{0,x,y,z}, can be expanded in terms of a single vector of an observational basis. Therefore payoffs corresponding to this game (Table 5) are single bimatrix pairs of the original classical game. Note that for any strategy of Player A, there is such a strategy of Player B, that the result of the quantum game is any pair of payoffs of the original game.

Having this matrix, one can now construct mixed Pauli strategies defined by quadruples of coefficients $\sigma^{X}={\left(\sigma_{\alpha }^{X}\right)}_{\alpha =0,x,y,z}$,

$$\Delta V_{X}\equiv \Delta \left(V_{X}\right)=\{\underset{\alpha =0,x,y,z}{\sum }\sigma_{\alpha }^{X}\hat{P_{\alpha }}\;|\;0\leq \sigma_{\alpha }^{X};\;\underset{\alpha =0,x,y,z}{\sum }\sigma_{\alpha }^{X}=1\},\;\;X=A,B.$$

Subsequently one can define a mixed quantum game in the EWL protocol $\Gamma_{EWL}^{mix}=\left(N,\;{\left\{\Delta V_{X}\right\}}_{X\epsilon N},\;{\left\{{\Delta \Pi }_{X}\right\}}_{X\epsilon N}\right)$, where the payoffs are defined by

$${\Delta \Pi }_{X}\left(\sigma^{A},\sigma^{B}\right)=\underset{\alpha ,\beta =0,x,y,z}{\sum }\sigma_{\alpha }^{A}\sigma_{\beta }^{B}\;\Pi_{X}\left(\hat{P_{\alpha }},\;\hat{P_{\beta }}\right)$$

Now it is possible to construct nontrivial Nash equilibria in mixed Pauli strategies. For the prisoner’s dilemma game from Table 1, the pair of strategies $\sigma^{A}=\left(\frac{1}{2},0,0,\frac{1}{2}\right)$ and $\sigma^{B}=\left(0,\frac{1}{2},\frac{1}{2},0\right)$ (or equivalently $\sigma^{\prime A}=\left(0,\frac{1}{2},\frac{1}{2},0\right)$ and $\sigma^{\prime B}=\left(\frac{1}{2},0,0,\frac{1}{2}\right)$) is a Nash equilibrium with payoffs $\left({\Delta \Pi }_{A},\;{\Delta \Pi }_{B}\right)=\left(\frac{5}{2},\;\frac{5}{2}\right)$. There is also a third equilibrium with a lower payoffs of $\left(\frac{9}{4},\;\frac{9}{4}\right)$ for a pair of strategies $\sigma {\prime \prime }^{A}=\sigma {\prime \prime }^{B}=\left(\frac{1}{4},\frac{1}{4},\frac{1}{4},\frac{1}{4}\right)$. Note that this quantum equilibrium gives both players a much higher payoff than the Nash equilibrium and the best correlated equilibrium, both yielding a payoff of (1,1).

Similarly, we can find a Nash equilibrium for battle of the sexes game from Table 2. Likewise the quantum PD, this game has no equilibrium in pure quantum strategies. One can check that, the highest payoffs of the game occur in two subgames defined by pairs of quantum strategies {$\hat{P_{0}}$,$\hat{P_{z}}$} and {$\hat{P_{x}}$,$\hat{P_{y}}$}. Therefore, one can be built two pairs of equilibria in mixed Pauli strategies $\sigma^{A}=\sigma^{B}=\left(\frac{1}{2},0,0,\frac{1}{2}\right)$ and $\sigma^{\prime A}=\sigma^{\prime B}=\left(0,\frac{1}{2},\frac{1}{2},0\right)$, that is, unlike the PD example, the Nash equilibrium is the case when Alice and Bob simultaneously play the same pair of strategies. The payoff for both pairs is then equal to $\left({\Delta \Pi }_{A},\;{\Delta \Pi }_{B}\right)=\left(\frac{5}{2},\;\frac{5}{2}\right)$, so exactly as for classical correlated equilibrium of this game.

For the chicken game the pair of Nash equilibrium mixed Pauli strategies is the same as in the prisoner’s dilemma $\sigma^{A}=\left(\frac{1}{2},0,0,\frac{1}{2}\right)$ and $\sigma^{B}=\left(0,\frac{1}{2},\frac{1}{2},0\right)$ (or equivalently $\sigma^{\prime A}=\left(0,\frac{1}{2},\frac{1}{2},0\right)$ and $\sigma^{\prime B}=\left(\frac{1}{2},0,0,\frac{1}{2}\right)$. In this equilibrium, drivers receive equal payoffs $\left({\Delta \Pi }_{A},\;{\Delta \Pi }_{B}\right)=\left(\frac{1}{2},\;\frac{1}{2}\right)$, the same, which provide the usual traffic lights and at the same time the best available in correlated equilibria. In the chicken game 2, the above pair of mixed Pauli strategies yields the payoffs $\left({\Delta \Pi }_{A},\;{\Delta \Pi }_{B}\right)=\left(3,\;3\right)$. Among equilibria giving both players equal payoffs, the above equilibrium gives the highest result and is better than the mixed strategy Nash equilibrium of the classical game (2$\frac{1}{2},\;2\frac{1}{2}$). It is however worse than maximal correlated equilibrium ($3\frac{1}{3},\;3\frac{1}{3}$). The comparison of the obtained results is presented in Table 6.

**Figure 2:**
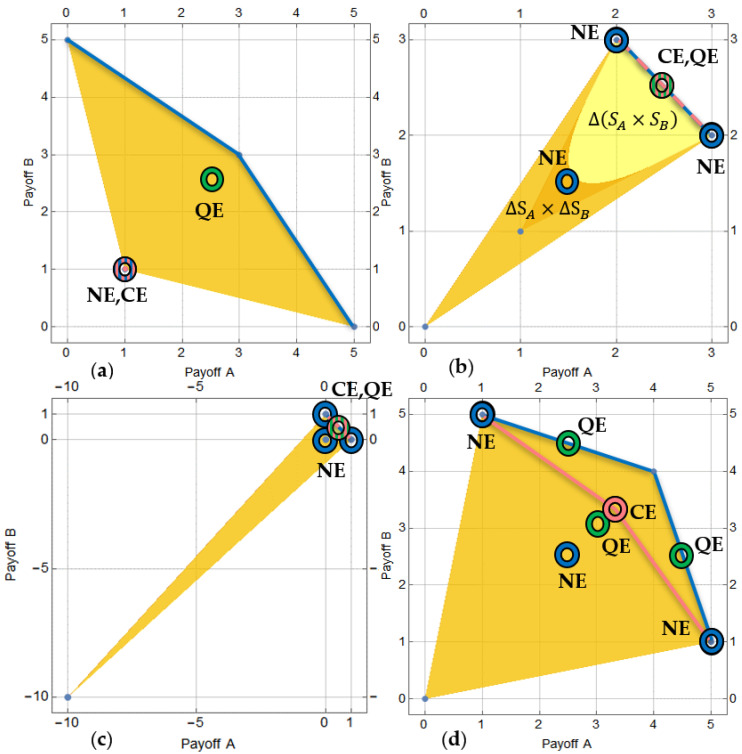
Probability distributions and equilibria of: (**a**) prisoner’s dilemma, (**b**) battle of the sexes, (**c**) chicken and (**d**) chicken 2 games defined by Table 1, Table 2, Table 3 and Table 4. Mixed strategies ${\Delta S}_{A}\times {\Delta S}_{B}$—golden area, probability distributions $\Delta \left(S_{A}\times S_{B}\right)\backslash {\Delta S}_{A}\times {\Delta S}_{B}$—yellow area, Pareto frontiers of $\Delta \left(S_{A}\times S_{B}\right)$—blue lines, Pareto frontiers of correlated equilibria—red lines, Nash equilibria—blue rings, symmetric correlated equilibria—red rings, quantum mixed strategy equilibria—green rings. Overlapping rings and dashed lines are filled with an appropriate mixed color pattern.

Interestingly, in the family of all mixed Pauli strategic equilibria, there is e.g., $\sigma^{A}=\left(\frac{1}{2},0,\frac{1}{2},0\right)$ and $\sigma^{B}=\left(\frac{1}{2},\frac{1}{2},0,0\right)$ or $\sigma^{\prime A}=\left(0,\frac{1}{2},0,\frac{1}{2}\right)$ and $\sigma^{\prime B}=\left(0,0,\frac{1}{2},\frac{1}{2}\right)$, which yields the payoff (2$\frac{1}{2}$, 4$\frac{1}{2}$), or symmetrically $\sigma^{\prime A}=\left(\frac{1}{2},\frac{1}{2},0,0\right)$ and $\sigma^{\prime B}=\left(\frac{1}{2},0,\frac{1}{2},0\right)$ or $\sigma^{\prime A}=\left(0,0,\frac{1}{2},\frac{1}{2}\right)$ and $\sigma^{\prime B}=\left(0,\frac{1}{2},0,\frac{1}{2}\right)$ with payoff (4$\frac{1}{2}$, 2$\frac{1}{2}$), i.e., of the sum of payoffs higher than in the correlated equilibrium. These equilibria are Pareto efficient. A graphical representation of all probability distributions, mixed strategies, Pareto frontiers, Nash equilibria, optimal symmetric correlated equilibria and the obtained quantum mixed equilibria is shown in Figure 2.

## 6. Conclusions

In this paper, we were looking for game solutions that would be closer to the Pareto-efficient results than classical game solutions. We took into account: the prisoner’s dilemma game, battle of the sexes and two versions of the chicken game. For most of these games (apart from PD), correlated equilibria are better than Nash equilibria. However, obtaining results in this way requires the introduction of an external device that correlates the actions of players. Such a device, sending signals to players, could be vulnerable to manipulation. Therefore, we proposed to use the quantum extension of games. We adopted the most common formalism of Eisert–Wilkens–Lewenstein quantum games, with 2-parameter strategy space introduced by Frąckiewicz and Pykacz. This parameterization scheme is invariant under isomorphic transformations of the input game. It has been shown that in this parameterization, the games under consideration have, in the mixed strategies, Nash equilibria much closer to Pareto-efficient solutions than the equilibria of classical games. These equilibria are comparable to correlated equilibria.

In the case of the prisoner’s dilemma, the Nash equilibrium of the quantum game corresponds to mixing with equal probability of cooperation and defection. Although this result is not Pareto-efficient, the players’ payoffs obtained in this way are better than the correlated equilibrium (equal to the Nash equilibrium) of the classical game. In the case of battle of the sexes, the quantum NE coincides with the best correlated equilibrium, it is fully fair for both partners and Pareto-efficient. For the chicken game, the Nash equilibrium of quantum game also coincides with the best Pareto optimal, correlated equilibrium. This solution is unattainable in classical mixed strategies. In the second version of the chicken game, the best equal solution obtained in mixed Pauli strategies is better than classical NE but worse than the one achievable in correlated equilibria. However, there are also Pareto efficient asymmetric equilibria with payoffs, the sum of which is greater than the sum for the correlated equilibrium.

In the conventional quantum game theory, mainly one-shot games have been studied. The nature of interpersonal interactions and the games people play are often repetitive processes. This leads to the formulation of the discussed optimization problems in the form of repeated (finitely or even infinitely) quantum games [61,62]. The results obtained by Aoki and Ikeda for the repeated quantum prisoner’s dilemma are very promising and set the direction for further research also on the games discussed in the present paper.

The question, whether quantum versions of games can contribute to solving practical economic situations, naturally arises. It is clear from this study, that solving games by means of a quantum strategy can give better results than conventional solutions. The advantage of quantum games lies in increasing the randomization of the game, which leads directly to results close to correlated equilibria, not available in classical games and that such a game can be played on a quantum computer—a tangible device that is resistant to external manipulation.

A general question can be asked: are there any connections between classical games and quantum phenomena? As a mathematical theory, classical games turn out to be a special case of quantum games. Do real classical games played by people every day have anything to do with physical quantum processes? The answer to this question may be surprising. A quantum phenomenon “suspected” of combining both realities is the collapse of the wave function. According to a recent hypothesis, the quantum fluctuations cause macroscopic phenomena that we consider random, such as, for example, tossing a coin or a die [63]. Moreover, every practical use of probability has its source in quantum phenomena. If this point of view were taken, any use of mixed strategy in a classical game would in fact be a quantum phenomenon.

In quantum games, an important element of the game mechanism is a quantum coherence, i.e., a definite phase relation between different states of the system. In practice, this means that the interaction between players is by nature a wave-like phenomenon, that has no equivalent in classical games. Problems with the decoherence of the wave function make it difficult to maintain two entangled qubits even at the level of strictly controlled experiments, taking place under extreme conditions of isolation from the environment. Building a quantum computer based on a register of many entangled qubits, subjected to unitary quantum gate operations and capable of solving practical problems or simulating quantum games with quantum algorithms is a real challenge. However, in recent years, we have seen more and more successful attempts to build such a computer and use it to implement quantum games.

## Figures and Tables

**Table 1:** The payoff matrix for the prisoner’s dilemma.

		Bob	
		*B*_0_	*B*_1_
**Alice**	***A*_0_**	(r, r)	(s, t)
	***A*_1_**	(t, s)	(p, p)

**Table 2:** The payoff matrix of battle of the sexes.

		Bob	
		*B*_0_	*B*_1_
**Alice**	***A*_0_**	(3, 2)	(1, 1)
	***A*_1_**	(0, 0)	(2, 3)

**Table 3:** The payoff matrix of the game of chicken.

		Driver B	
**Driver A**		***B*_0_**	***B*_1_**
	***A*_0_**	(0, 0)	(0, 1)
	***A*_1_**	(1, 0)	(−10,−10)

**Table 4:** The payoff matrix of the game of chicken 2.

		Player B	
**Player A**		***B*_0_**	***B*_1_**
	***A*_0_**	(4, 4)	(1, 5)
	***A*_1_**	(5, 1)	(0, 0)

**Table 5:** The payoff matrix of Pauli strategies in the EWL scheme.

		Player B			
		**$\hat{P_{0}}$**	**$\hat{P_{x}}$**	**$\hat{P_{y}}$**	**$\hat{P_{z}}$**
**Player A**	$\hat{P_{0}}$	$\left(v_{00}^{A},v_{00}^{B}\right)$	$\left(v_{01}^{A},v_{01}^{B}\right)$	$\left(v_{10}^{A},v_{10}^{B}\right)$	$\left(v_{11}^{A},v_{11}^{B}\right)$
	$\hat{P_{x}}$	$\left(v_{10}^{A},v_{10}^{B}\right)$	$\left(v_{11}^{A},v_{11}^{B}\right)$	$\left(v_{00}^{A},v_{00}^{B}\right)$	$\left(v_{01}^{A},v_{01}^{B}\right)$
	$\hat{P_{y}}$	$\left(v_{01}^{A},v_{01}^{B}\right)$	$\left(v_{00}^{A},v_{00}^{B}\right)$	$\left(v_{11}^{A},v_{11}^{B}\right)$	$\left(v_{10}^{A},v_{10}^{B}\right)$
	$\hat{P_{z}}$	$\left(v_{11}^{A},v_{11}^{B}\right)$	$\left(v_{10}^{A},v_{10}^{B}\right)$	$\left(v_{01}^{A},v_{01}^{B}\right)$	$\left(v_{00}^{A},v_{00}^{B}\right)$

**Table 6:** Comparison of the best symmetric game results.

Game Name	Table Nos.	Best Symmetrical Pareto-Efficient Payoffs in $\Delta \left(S_{A}\times S_{B}\right)$	Best Symmetrical Payoffs for the		
			Nash Equilibrium	Correlated Equilibrium	NE in Mixed Pauli Strategies
**Prisoner’s dilemma**	**1**	3	1	1	212
**Battle of the sexes**	**2**	212	112	212	212
**The game of chicken**	**3**	12	0	12	12
**The game of chicken 2**	**4**	4	212	313	3

## Data Availability

Not applicable.
